# Case Report: Langerhans Cell Sarcoma With Intracranial and Extracranial Communication on the Left Frontal Bone

**DOI:** 10.3389/fsurg.2022.882694

**Published:** 2022-06-07

**Authors:** Shan Xie, Zhilin Shao, Dongqi Shao, Bang Du, Yi Han, Yu Li, Binbin Zhang, Xialin Zheng, Zhiquan Jiang

**Affiliations:** ^1^ Department of Neurosurgery, The First Affiliated Hospital of Bengbu Medical College, Bengbu, China; ^2^ Department of Emergency Surgery, The First Affiliated Hospital of Bengbu Medical College, Bengbu, China

**Keywords:** langerhans cell sarcoma, left frontal, pathological diagnosis, surgical resection, malignant tumor

## Abstract

**Background:**

Langerhans cell sarcoma (LCS) is an extremely rare type of malignant tumor that originates from Langerhans cells (LC). It is characterized by the malignant proliferation and dissemination of LC and is extremely invasive, with rapid progression and a poor prognosis. Treatment includes resection of lesions, radiotherapy, chemotherapy, immunotherapy, and transplantation of hematopoietic stem cells. However, a unified and optimized treatment plan is lacking, and individualized treatment is accepted.

**Case presentation:**

We report an 18-year-old man with intracranial and extracranial communicative LCS that occurred in only the left forehead without metastasis to other regions. Clinical and hematological data were normal. We undertook complete resection of diseased tissue, which was pathologically examined. Staining (hematoxylin and eosin) showed positivity for cluster of differentiation (CD)1a (++), S-100 protein (++), P53 (++), CD68 (+), cyclin D1 (+), cyclin A (+), cyclin B1 (+), IGF2BP3 (+), and Ki-67 (45%–50%). Recurrence or metastasis were not observed after long-term follow-up.

**Conclusion:**

LCS is a rare malignant tumor, and one that occurs with intracranial and extracranial communication is even rarer. Active adoption of an individualized treatment plan is crucial.

## Introduction

In health, Langerhans cells (LC) are found in the basal endothelial cells of the skin and mucous membranes. LC are specifically differentiated dendritic cells during an immature stage of development. Once activated, LC present specific antigens to T cells and migrate to lymph nodes via lymphatic vessels.

An LC tumor originates from LC and maintains its immunophenotype and ultrastructural characteristics. According to the degree of atypical behavior and clinical invasiveness, swollen LC tumors are classified according to the histiocyte system and are divided into two main subcategories: Langerhans cell hyperplasia (LCH) and Langerhans cell sarcoma (LCS) (1).

LCS is an extremely rare tumor characterized by the malignant proliferation and dissemination of LC. It is considered a high-grade variant of Langerhans cell histiocytosis, which can be a primary variant or one that develops from Langerhans cell histiocytosis (2). LCS was first reported by Wood et al. in 1984 (3). A literature search from the PubMed database (https://pubmed.ncbi.nlm.nih.gov/) reveals that ∼30 cases have been reported. Clinical cases of LCS that occur in the head are even rarer.

## Case Presentation

### Clinical History and Laboratory Findings

An 18-year-old man was admitted to hospital with a chief complaint of a lump on his left forehead, which had been detected during physical examination. He stated that the lump appeared without obvious inducement 1 month previously. It was flat mound-shaped, protruded the skin, and was approximately 2 × 3 cm in size. It had clear borders, a tough texture, and was non-movable. It was not red, swollen, painful, and local skin ulcers or sinus formation were not observed.

Body temperature was normal at the time of hospital admission. Skin abnormalities on other parts of the body were not found during physical examination. He had no swelling of the liver, spleen, or lymph nodes. He had no history of skin diseases or other diseases.

A routine blood test upon hospital admission showed a hemoglobin level of 156 g/L, as well as counts (×10^12^/L) of 5.47 for red blood cells, 5.55 for white blood cells, 2.99 for neutrophils, and 120 for platelets. Coagulation function, liver function, kidney function, electrolyte levels and the level of C-reactive protein were normal.

### Computed Tomography (CT) and Magnetic Resonance Imaging (MRI) of the Head

**Figure 1:**
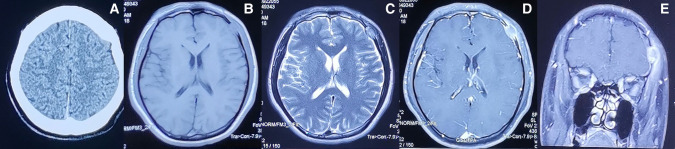
Preoperative imaging information of LCS (**A**) Computed tomography and (**B**–**E**) magnetic resonance imaging findings of the intracranial and extracranial communication lesions in the left forehead.

CT of the head after hospital admission showed that the left frontal bone was damaged, but an obvious abnormal-density shadow was not seen in brain parenchyma. The midline structure was centered, and the sub-tentorial cerebellum/brainstem was normal. MRI of the head showed the left frontal bone to be destroyed and to have grown inwards and outwards into a flat mound-like mass. Furthermore, the T1-weighted signal was slightly longer and the T2-weighted signal was altered in the plain MRI scan. The signal was uniform, and the inner and outer plates of the skull were incomplete. Contrast-enhanced MRI showed obvious enhancement of the lesion at approximately 1.6 × 2.0 cm. The adjacent dura mater was thickened, the brain parenchyma was slightly compressed, the soft tissues of the scalp were swollen outwards, and the tumor was strengthened (Figure 1). We carried out whole-body positron emission tomography (PET)–CT preoperatively and found no metastatic lesions. CT of the chest and abdomen were reviewed postoperatively. Metastasis was not found, so radiotherapy or chemotherapy were not initiated.

### Intraoperative Situation

Following the induction of general anesthesia, the patient was placed in supine with the head tilted to the right. An arc-shaped incision centered on the mass was made according to the skin level. The lesion was located under muscle, had a soft texture, as well as a clear boundary that separated gradually along the skull surface. The skull had been invaded, part of the defect, enlarged along the edge of the defect to bite off the invaded skull, the tumor was tightly adhered to the dura, and the dura was completely removed from the edge of the tumor. The defect in the dura was repaired using an artificial dura mater. The skull defect was repaired using a titanium mesh and by suturing each layer of the scalp.

### Pathology

**Figure 2:**
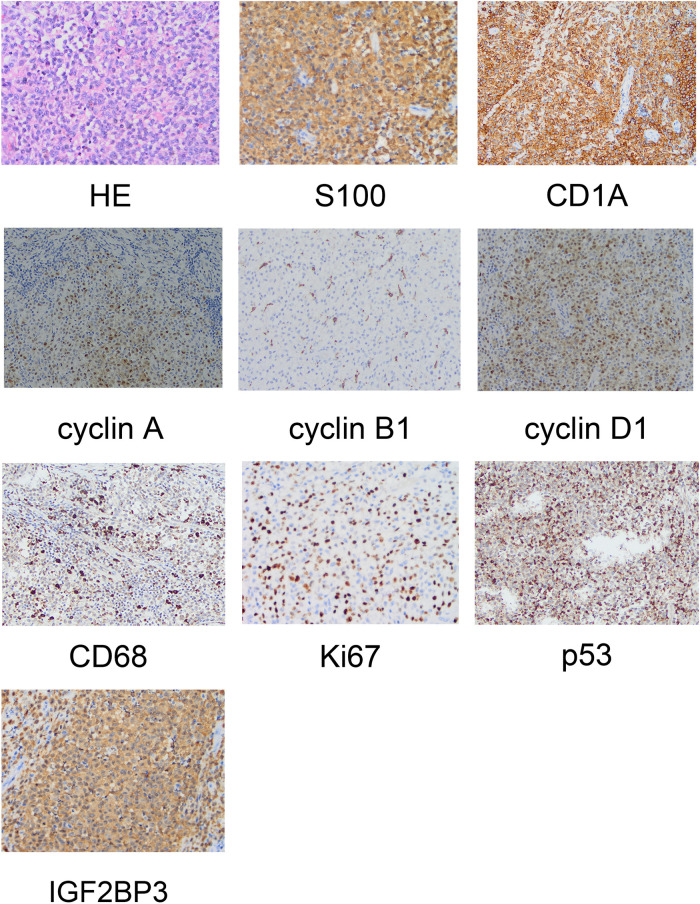
Pathological examination revealing the morphology of Langerhans cell sarcoma and positive immunohistochemical markers.

The intraoperative specimens obtained were examined. Staining (hematoxylin & eosin) showed left frontal LCH, active proliferation of tumor cells in the focal area, and visible nucleoli. We suspected that the malignant tumor had become LCS. Immunohistochemical analyses showed positivity for cluster of differentiation (CD)1a (++), S100 protein (++), P53 (++), CD68 (+), cyclin D1 (+), cyclin A (+), cyclin B1 (+), IGF2BP3 (+), and Ki-67 (45%–50%) (Figure 2).

### Outcome and Follow-Up

**Figure 3:**
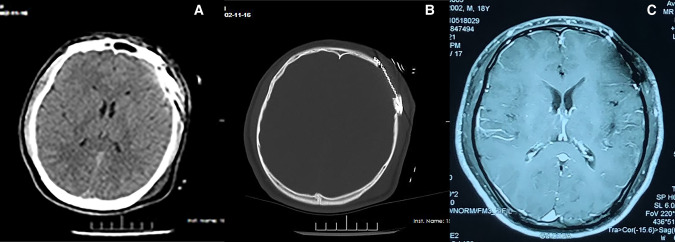
Imaging information were followed up one year after surgery of LCS (**A,B**) CT (**C**) MRI.

Postoperatively, the patient was discharged from hospital. Gradually, his life returned to normal (including limb movement). Consultation at 1-year follow-up involved re-examination of the head using CT and MRI: recurrence and metastasis had not occurred (Figure 3).

## Discussion and Conclusions

LCS can occur at any age, with onset ranging from age 10 years to 81 years and a median age of 51.5 years. The male:female ratio is approximately 1.3:1, with a slightly higher prevalence in females than males (4). The cause and pathogenesis of LCS are not known, but reports suggest that they are related to uncontrolled regulation of the immune system and a proliferation disorder of LC (5). Some cases of LCS may be associated with other tumors, such as follicular lymphoma, adenocarcinoma, and germ-cell tumors, which also occur in patients with schizophrenia who have undergone long-term treatment (6–8). Recently, there have been reports of LCH or LCS following organ (e.g., liver) transplantation, which may be related to long-term use of immunosuppressive agents (9). The clinical manifestations of LCS are similar to those of Langerhans cell histiocytosis, but are more serious. LCS can occur in lymph nodes, skin, liver, spleen, lung, bone, and soft tissue.

There are no reports of LCS originating from intracranial or extracranial communication. Moreover, a unified standard for the diagnosis and treatment of LCS is lacking. The pathological diagnosis is based mainly on positive staining of certain biochemical markers.

Specimens obtained from patients with LCS are subject to pathological examination. Common findings include the characterization of LC: as oval or coffee bean-like cells; having nuclear grooves; exhibiting folding, depressions, or lobules; possessing fine chromatin; comprising vague nuclei with a low mitotic ratio. In contrast, LCS cells are large with markedly abnormal chromatin, deformation, clear nucleoli, and a high mitotic ratio of >50/HPF. LCs and LCS cells have a distinct morphology. In our patient, immunohistochemical analyses showed positivity for CD1 and S100 protein.

Inhibition of p53 expression during DNA damage inhibits cell proliferation and induces apoptotic cell death (10). This case report supports the notion that p53 is involved in the pathogenesis of histiocytic sarcoma. p53 protein can be detected readily in the nuclei of tumor cells in some patients and we demonstrated that expression of cyclins A, B1, and D1 was significantly higher in LCS cells. IGF2BP3 is expressed in malignant cells with aggressive or proliferative phenotypes, and is thought to be related to expression of cyclins A, B1, and D1 (11), It has been reported that IGF2BP3 may be the key factor to distinguish LCH from LCS, and may become a prognostic marker. In our patient, IGF2BP3 showed high expression (12).

Howard and colleagues reported that treatment and management of LCS vary greatly (4): 52% of patients were treated using a single method, 42% of patients were treated with combination therapy, and the remaining four patients (6%) received no treatment. Treatments can be divided into three categories: chemotherapy, surgery, or combined radiotherapy. Six percent of patients received bone-marrow transplantation as additional treatment.

Wang et al. (13) reported a patient with multifocal cutaneous lesions involving bilateral inguinal regions and wais, The patient underwent six cycles with cyclophosphamide, oncovin, prednisone along with radiotherapy after surgery, but the patient died. Stephanie et al. (14) treated a LCS patient with radiotherapy (45.6 Gy) after surgery, whereas Li et al. provided six cycles of systemic CHOP: the patients survived without suffering recurrence. Importantly, many researchers have undertaken resection only in a single patient with LCS, and these patients have reported good outcomes. We undertook preoperative whole-body PET–CT and found no metastatic lesions, CT of the chest and abdomen were reviewed postoperatively: metastasis was not detected.

In terms of the prognosis, patients with single-organ disease have a clear advantage over those with damage to other organs. However, because of the rarity of LCS, drawing definitive conclusions is challenging. Nevertheless, multimodal therapies appear to be the most efficacious. Surgery is the most efficacious if a clear lesion edge can be identified (15). Furthermore, adjuvant treatment should not be delayed if treatment is planned. A consensus on the chemotherapy regimen is lacking, so further research and the development of guidelines are important. Bone-marrow transplantation appears to be a reliable treatment method, but patient selection, toxicity, and tolerability issues have been documented. Although abnormal expression of various cell-surface markers has been reported, the pathogenesis as well as prognostic and therapeutic importance of LCS is not clear. Therefore, further research on this rare disease is necessary. Deeper understanding of the diagnosis and treatment of LCS will provide significant benefits.

## Data Availability

The original contributions presented in the study are included in the article/Supplementary Material, further inquiries can be directed to the corresponding author/s.
